# Monogenic Autoinflammatory Diseases with Mendelian Inheritance: Genes, Mutations, and Genotype/Phenotype Correlations

**DOI:** 10.3389/fimmu.2017.00344

**Published:** 2017-04-03

**Authors:** Davide Martorana, Francesco Bonatti, Paola Mozzoni, Augusto Vaglio, Antonio Percesepe

**Affiliations:** ^1^Unit of Medical Genetics, University Hospital of Parma, Parma, Italy; ^2^Department of Medicine and Surgery, University Hospital of Parma, Parma, Italy; ^3^Unit of Nephrology, University Hospital of Parma, Parma, Italy

**Keywords:** autoinflammatory diseases, hereditary periodic fevers, familial Mediterranean fever, mevalonate-kinase deficiency, tumor necrosis factor receptor-associated periodic syndrome, cryopyrinopathies, inflammasome, whole exome sequencing

## Abstract

Autoinflammatory diseases (AIDs) are a genetically heterogeneous group of diseases caused by mutations of genes encoding proteins, which play a pivotal role in the regulation of the inflammatory response. In the pathogenesis of AIDs, the role of the genetic background is triggered by environmental factors through the modulation of the innate immune system. Monogenic AIDs are characterized by Mendelian inheritance and are caused by highly penetrant genetic variants in single genes. During the last years, remarkable progress has been made in the identification of disease-associated genes by using new technologies, such as next-generation sequencing, which has allowed the genetic characterization in undiagnosed patients and in sporadic cases by means of targeted resequencing of a gene panel and whole exome sequencing. In this review, we delineate the genetics of the monogenic AIDs, report the role of the most common gene mutations, and describe the evidences of the most sound genotype/phenotype correlations in AID.

## Introduction

The term autoinflammatory disease (AID) was proposed in 1999 to describe a group of disorders of the innate immune system characterized by recurrent episodes of inflammation without a known origin (1). AIDs are frequently caused by genetic mutations in genes encoding proteins involved in the pathways of the inflammasome, with a crucial role of proinflammatory interleukin-1 (IL-1), which is an important cytokine of the systemic inflammatory response.

Autoinflammatory diseases have in the most of cases a genetic background, with highly penetrant mutations of single genes, but in some cases are polygenic, with a strong environmental influence that can modulate the phenotype (2).

The first AID described was the familial Mediterranean fever (FMF), which is also the most prevalent AID in the world. After FMF, other two AIDs were described: TNF-receptor associated periodic syndrome (TRAPS) (1) and hyperimmunoglobulinemia D with periodic fever syndrome [hyper-IgD syndrome (HIDS)/mevalonate kinase (MVK)] (3–5). These three forms of AID were grouped in the hereditary periodic fever syndromes, because they share fever episodes. After these, other AIDs were identified, such as familial cold autoinflammatory syndrome (FCAS), Muckle–Wells syndrome (MWS), and neonatal onset multisystem inflammatory disease (NOMID), also known as chronic infantile neurologic cutaneous and articular (CINCA) syndrome, then grouped in the cryopyrinopathies. These disorders are also known as cryopyrin-associated periodic syndromes (CAPS).

Recently, mutations in single genes involved in IL-1 processing have been demonstrated in the deficiency of IL-1 receptor antagonist (DIRA) (6). After this, evidences of drugs able to block IL-1 in the Majeed syndrome (MS), an AID bone disease with clinical similarities to DIRA, demonstrated the pivotal role of IL-1 in this disorder (7). The same drug was used for cryopyrinopathies and DIRA, further expanding its use to other monogenic AID disorders. Other granulomatous disorders are characterized by typical granulomatous formations: Blau syndrome (familial juvenile systemic granulomatosis) is characterized by granulomatous inflammation of joint, skin, and uvea.

In this review, we will focus only on monogenic AIDs, which are mostly represented by early-onset conditions and a clear pattern of autosomal dominant or recessive transmission, at least in some of the families (Table 1) and the clinical significance of exonic variants according with pathogenic criteria (Table 2). The review will analyze the evidences about the mutations in the genes involved in the pathogenesis of the disease and the genotype/phenotype correlations (8).

**Table 1:** **Classification of monogenic autoinflammatory diseases (AIDs)**.

Disorder (abbreviation)	#OMIM	*Gene* (Locus)	Protein involved	Inheritance
Familial Mediterranean fever	249100	*MEFV* (16p13.3)	Pyrin (marenostrin)	Autosomal recessive
Hyper-IgD syndrome	260920	*MVK* (12q24.11)	Mevalonate kinase	Autosomal recessive
Mevalonate kinase deficiency	260920	*MVK* (12q24)	Mevalonate kinase	Autosomal recessive
Tumor necrosis factor receptor-associated periodic syndrome	142680	*TNFRSF1A* (12p13)	Tumor necrosis factor receptor type-1	Autosomal dominant
Familial cold autoinflammatory syndrome (FCAS)	120100	*NLRP3* (1q44)	Cryopyrin	Autosomal dominant
Muckle–Wells syndrome	191900	*NLRP3* (1q44)	Cryopyrin	Autosomal dominant
Neonatal onset multisystem inflammatory disease	607115	*NLRP3* (1q44)	Cryopyrin	Autosomal dominant
Deficiency of interleukin (IL)-1 receptor antagonist	612852	*IL1RN* (2q)	IL-1 receptor antagonist	Autosomal recessive
Blau syndrome	186580	*NOD2/CARD15* (16q12.1-13)	Nucleotide-binding oligomerization domain-containing protein 2	Autosomal dominant
Deficiency of the IL-36 receptor antagonist	614204	*IL36RN* (2q14)	IL-36 receptor antagonist	Autosomal recessive
Chronic atypical neutrophilic dermatosis with lipodystrophy and elevated temperature syndrome	256040	*PSMB8* (6p21)	Inducible subunit β of the proteasome	Autosomal recessive
Majeed syndrome	609628	*LPIN2* (18p11.31)	Lipin 2	Autosomal recessive
CARD14-mediated pustular psoriasis	177900	*CARD14* (17q25.3)	Caspase recruitment domain family member 14	Autosomal dominant
NLRP12-autoinflammatory disease	609648	*NLRP12* (19q13.42)	Monarch 1	Autosomal dominant
Pyogenic arthritis, pyoderma gangrenosum, and acne syndrome	604416	*PSTPIP1* (15q24-25)	CD2 antigen-binding protein 1	Autosomal dominant
Deficiency of adenosine deaminase 2	615688	CECR1 (22q11.1)	Adenosine deaminase 2	Autosomal recessive
STING-associated vasculopathy	615934	*TMEM173* (5q31.2)	Transmembrane protein 173	Autosomal dominant
TNFRSF11A-associated disease	603499	*TNFRSF11A* (18q21.33)	Tumor necrosis factor receptor 11A	Autosomal dominant
NLRC4-associated diseases (NLRC4-MAS, SCAN4, NLRC4-FCAS)	606831	*NLRC4* (2p22.3)	NLR family CARD domain-containing Protein 4	Autosomal dominant
Sideroblastic anemia, B-cell immunodeficiency, periodic fevers, developmental delay	616084	*TRNT1* (3p26.2)	CCA-adding enzyme	Autosomal recessive
Monogenic form of systemic juvenile idiopathic arthritis	613409	*LACC1* (13q14.11)	Laccase (multicopper oxidoreductase) domain containing 1	Autosomal recessive

**Table 2:** **Clinical significance of exonic variants according with the pathogenic criteria of ClinVar database (https://www.ncbi.nlm.nih.gov/clinvar/) for genes *MEFV, MVK, TNFRSF1A*, and NLRP3**.

Gene	Clinical significance					
	Conflicting interpretation	Benign	Likely benign	Uncertain significance	Likely pathogenic	Pathogenic
*MEFV*	Leu110Pro	Leu110Pro	Glu148Gln	Ser6Arg	Gly304Arg	Glu148Gln
	Glu148Gln	Glu148Gln	Arg202Gln	Val33Leu	Pro369Ser	Glu148Val
	Glu148Val	Gly196Trp	Gly304Arg	Arg42Trp	Arg408Gln	Glu167Asp
	Gly196Trp	Arg202Gln	Arg408Gln	Asn78Ile	Met680Ile	Pro180Gln
	Gly304Arg	Pro369Ser	Ile591Thr	Asn78Ser	Lys695Arg	Thr267Ile
	Pro369Ser	Arg408Gln		Glu84Gln	Ala744Ser	Glu276Ter
	Arg408Gln			Glu93Gln		Leu367Val
	Ile591Thr			Gln97Ter		Pro369Ser
	Lys695Arg			Asp103His		His404Arg
				Ser108Arg		Thr577Asn
				Leu110Pro		Arg478Gln
				Pro115Thr		His478Tyr
				Asp122Gly		Phe479Leu
				Gly136Glu		Ile591Thr
				Gln146Ter		Arg653His
				Pro147Ala		Met680Ile
				Glu148Gln		Gly687Asp
				Glu148Alafs		Tyr688Ter
				Glu148Val		Ile692del
				Arg151Thr		Met694Val
				Glu163Gln		Met694del
				Ala171Thr		Lys695Arg
				Gln172Pro		Val726Ala
				Pro183Thr		Ala744Ser
				Ala193Thr		Arg761His
				Gly196Trp		
				Glu230Lys		
				Lys266Glu		
				Gly304Arg		
				Thr309Met		
				Ala311Val		
				Arg314His		
				Gly320Ala		
				Arg329His		
				Ser339Phe		
				Arg348His		
				Gln356Glu		
				Pro369Ser		
				Pro383Arg		
				Arg480Gln		
				Gln440Glu		
				Glu446Ala		
				Lys447Asn		
				Ala457Val		
				Arg461Gln		
				Val469Ala		
				Asp505Tyr		
				Arg579His		
				Ile591Thr		
				Asn599Asp		
				Lys625Gln		
				Pro630Alafs		
				Arg653Cys		
				Gly678Glu		
				Lys695Arg		
				Pro714Leu		
				Lys716Glu		
				Phe743Leu		
				Ile772Val		
				Pro780Thr		
*MVK*	Val80Ile	Arg19His	Arg19Gln	Pro11Leu	Ile268Val	Met1Thr
		Ser52Asn	Ser52Asn	Val15Ala		Leu6Glyfs
			Val180Ile	Cys21Ser		His20Pro
				Leu27Phe		Gly25Trpfs
				Val80Ile		Leu41Pro
				Cys101Tyr		Tyr116His
				Arg106His		Gly140Argfs
				Ala111Thr		Ala141Glyfs
				Pro200Ser		Ala148Thr
				Pro286Leu		Pro165Leu
				Gln302Ter		Pro167Leu
				Arg388Gln		Trp188Ter
						Gly202Arg
						Val203Ala
						Arg215Ter
						Leu255Pro
						Ile268Thr
						Asn301Thr
						Val310Met
						Ala334Thr
						Phe365Ser
						Val377Ile
						Arg388Ter
*TNFRSF1A*	Arg121Gln	None	Pro75Leu	Leu96Pro	Asp41Glu	Cys59Arg
				Val112Met	Phe89Leu	Cys59Ser
				Arg121Gln	Asn94Lys	Cys62Gly
				Val124Met	Arg106Gln	Cys62Tyr
				Asn145Ser		Thr79Met
				Glu178Lys		Cys81Phe
				Ile199Thr		Cys99Ser
				Pro269Arg		Cys99Arg
				Pro275Ser		Cys117Arg
				Pro412Ala		Cys117Tyr
				Ser452Arg		Arg121Pro
						Arg121Gln
*NLRP3*	Val198Met	Gln705Lys	Met70Thr	Ala67Glu	Leu305Pro	Val198Met
	Pro315Leu		Val72Met	Ala77Glu	Arg488Lys	Arg206Trp
	Arg488Lys		Ser196Asn	Ala77Val	Gln602Arg	Asp303Asn
	Gln705Lys		Val198Met	Arg100Cys		Glu304Lys
	Ser728Gly		Pro315Leu	Lys131Arg		Phe309Ser
	Thr954Met		Arg488Lys	Arg137His		Thr348Met
			His713Leu	Asn165Ser		Ala352Val
			Ser728Gly	Thr195Met		Leu353Pro
			Thr954Met	Val198Met		Thr405Pro
				Asp212Asn		Ala439Val
				Ala225Val		Gly569Arg
				Gln250Arg		Gly571Arg
				Pro315Leu		Phe573Ser
				Lys357Arg		Glu627Gly
				Thr435Ala		Tyr859Cys
				Leu447Phe		
				Gly456Glu		
				Lys615Asn		
				Gln705Lys		
				Ser728Gly		
				Gly769Ser		
				Leu800Met		
				Gly811Ser		
				Leu832Ile		
				Ala848Pro		
				Ala873Thr		
				Lys880Glu		
				Thr915Met		
				Thr923Ala		
				Lys930Asn		
				Thr954Met		
				Cys990Ser		
				Cys998Ser		
				Lys1015Glu		

### Familial Mediterranean Fever

Familial Mediterranean fever is the most common AID, which is inherited as autosomal recessive disease, although features of autosomal dominant pattern of transmission have been demonstrated in several families (9). This different pattern might have conferred an evolutionary advantage in the resistance to an endemic pathogen; in fact, Clostridium, Yersinia, *Vibrio parahaemolyticus* VopS, *Histophilus somni* IbpA, Burkholderia, and other microbes that modify RhoGTPases are able to stimulate pyrin inflammasome. Pyrin play a role in sensing pathogen modification and inactivation of Rho GTPases (10, 11). Furthermore, in some populations, as Sephardic Jews, Turks, Arabs, and Armenians, the carrier rate for a mutant *MEFV* allele is high, ranging from 1/3 to 1/6; this represents the highest carrier rates reported for an autosomal recessive disorder.

To estimate prevalence in FMF is difficult, because of the wide range of differences in areas of diffusion of the disease. In particular, the most affected patients belong to Middle Eastern living around the Mediterranean Sea areas.

The causing gene, *MEFV*, was identified in 1997 by two International Consortia, who named the encoded protein pyrin/marenostrin (11, 12), an intracellular regulator of IL-1 production (13, 14). The disease-causing mutations spread all over the gene, even if the exon 10 carries the most typical and severe mutation; in fact, this exon encodes for the B30.2/SPRY domain at the C-terminal end of pyrin, which is demonstrated to interact with the protein caspase 1 (15). Although five mutations represent more than 85% of all disease-associated mutations, many other mutations with different clinical penetrances have been reported so far (in the Infevers website more than 300 mutations are described) (16). Genetic test can support clinical diagnosis, confirming the presence of two mutations in the *MEFV* gene, although patients with a heterozygous mutation can show clinical pictures of FMF, even if with an incomplete phenotype (17).

In fact, there are evidences that the mutation in the second allele is not demonstrated in 20–25% of the patients with the clinical picture of FMF and a positive response to colchicine therapy (18). The reduced diagnostic accuracy of the genetic tests in terms of mutation finding is common to most of the genes studied for diagnostic purposes, since the diagnostic yield of the tests is never complete, due to factors like genetic heterogeneity, incorrect diagnosis, or phenocopies. When a heterozygous is found, the most obvious hypothesis is that the second disease allele lies in other genic regions not explored by the test (i.e., deep intronic regions); however, the second mutation has not been found also in studies analyzing the promoter and intron regions of the *MEFV* gene. In FMF, however, the evidence of autosomal dominant transmission, segregating a heterozygous mutation (17), or a complex allele (personal data) is consistent with a dosage effect, which is dependent on the type of the mutation, in analogy to models already described in other disease genes (i.e., *GJB2*, in which biallelic mutations cause autosomic recessive nonsyndromic deafness and dominant mutations in specific domains of the gene cause syndromic forms of deafness with palmoplantar keratoderma). Another possible pathogenetic model for the dominant forms could involve the interaction of genetic and environmental factors in the pathogenesis of the disease, in analogy with hemochromatosis, in which heterozygotes for the C282Y in the *HFE* gene are bona fide healthy carriers unless other factors like alcohol or viral infections induce the onset of the clinical phenotype by contributing to the accumulation of iron in the liver. In a genomic study of 22 Belgian individuals, 12 of whom had clinical pictures of AID, the pattern of Mendelian inheritance was autosomal dominant. The phenotype, different from FMF, was characterized by childhood-onset recurrent episodes of neutrophilic dermatosis, fever, elevated acute-phase reactants, arthralgia, and myalgia/myositis. The disease was named pyrin-associated autoinflammation with neutrophilic dermatosis (PAAND). Genomic analysis revealed a mutation in the *MEFV* gene, S242R. This mutation causes a loss of a binding motif in the pyrin protein different from the B30.2/SPRY domain. Interestingly, the loss of the S242 domain was observed in bacterial effectors able to activate the pyrin inflammasome, such as Clostridium difficile toxin B (TcdB). As a result, the S242R mutation has the same effect of pathogen sensing, acting as a trigger of the inflammasome activation and IL-1b production. Based on this fact, the affected patients were successfully treated with therapy targeted on IL-1b, resolving autoinflammation and neutrophilic dermatosis (19).

Another intriguing aspect in FMF and, more in general AID, is the different influences of certain mutations or polymorphisms on the phenotype. For example, an E148Q mutation is sometimes referred to as a functional polymorphism because of the high carrier rate (more than 10%) and the lack of phenotype in some homozygous patients. However, some patients may have severe disease expression as well. Thus, the suggestions put forward for the carrier state may apply to these states as well.

Environmental factors have been investigated in relation with disease severity in FMF (19). The strongest association correlated with amyloidosis was with the country of origin, not with the genotype. Another interesting study showed that Turkish children who are born and live in Turkey have a higher disease severity score compared with Turkish children living in Germany (20); this study demonstrated that the different patterns of infections influence the expression of the phenotype acting as a trigger of the weak innate immune pathway via pathogen-recognition factors.

In order to search for possible mutation in the *MEFV* gene, a multiplex ligation-dependent probe amplification was setup in 216 FMF patients (21). No copy number variants were identified, suggesting that deletion/duplication is not a mutational mechanism in MEFV. The possible functional explanation relies on the pyrin function, which is crucial in the immune surveillance, even if with genetic variants such as point mutations and functional polymorphisms able to modulate its function.

An interesting study aimed at investigating pyrin function during primate evolution analyzed the domain which contains the most MEFV mutations, named ret finger protein domain. Amino acids involved in MEFV mutations (653, 680, 681, 726, 744, and 761 residues) in human are frequently present as wild type in other primates. In some cases, the mutant may be considered the reappearance of an ancestral amino acid state. As a result, an episodic positive selection was postulated. These changes in pyrin sequence could be caused by selective pressures driven from environmental agents (22).

Regarding the therapy, both a single mutation and two mutations, supported by clinical pictures of FMF, support the use of colchicine (23). Amyloidosis type AA is frequently correlated with the MEFV mutation M694V and the SAA1.1/SAA1.1 genotype (24).

In analogy with most of the disorders with pleiotropic expression, also for FMF, strict clinical criteria have been proposed for the diagnosis (25), taking into account the phenotypic manifestations (the recurrence of fever with serositis), the histological picture (idiopathic AA amyloidosis), and the response to therapy (in typical FMF, the colchicine test results in a favorable response). Two major criteria are necessary for a definite diagnosis of FMF. Recurrent fever without serosal involvement, cutaneous, erysipelas-like manifestations, and a positive family history for the disease in a first-degree relative are considered minor criteria for the lower specificity of the features and take part in the algorithm for diagnosis only if a major criterion is present. The presence of two mutations in *MEFV* gene is generally achieved in patients fulfilling the clinical criteria, but also, to a much lesser extent, also in subjects with atypical phenotypic features, whereas also heterozygous mutation carriers can suffer from an incomplete and even typical disease (17). For all these reasons, the detection of a single heterozygous mutation, in the presence of clear clinical symptoms, appears to be a sufficient prerequisite for a colchicine trial (23).

### HIDS/Mevalonate Kinase Deficiency (MKD)

Periodic fever associated with MKD was originally identified in 1984 in patients of Dutch ancestry; they reported recurrent attacks of fever of unknown origin and a high serum IgD level (26). After this first description, the disease was named Dutch fever or HIDS. After the first report, MKD was then described in other European countries around the Mediterranean basin (27) and Asia (28).

Because of the low sensitivity and specificity of the raised IgD serum levels, the term HIDS has been replaced by periodic fever associated with MKD after the discovery of the causing mutations in the *MVK* gene (OMIM *251170) located on chromosome 12q24 (29).

Mevalonate kinase deficiency, also known as hyperimmunoglobulinemia D syndrome (OMIM 260920) is characterized by an autosomal recessive Mendelian inheritance pattern (5) and is allelic to another disorder, mevalonic aciduria (MA, OMIM 610377), characterized by a very low activity of the enzyme MVK.

As in other AIDs, the carrier rate of 1/350 in normal population allowed to hypothesize a selective advantage for heterozygous carriers; a possible explanation postulates that countries with a diet at high consumption of saturated animal fats rich in cholesterol could have selected heterozygous carriers of the most frequent *MVK* mutations (30); nevertheless, this theory has not been demonstrated and other possible explanations are possible.

So far more than 130 substitutions or deletions of the *MVK* gene have been reported (16), even if a small number of mutations (V377I, I268T, H20P, and P167L) represent the 71.5% of the whole mutation spectrum in MKD patients (29).

Several genotype–phenotype associations have been described. The most common mutation is the V377I variant, which is associated with a mild phenotype of MKD and some residual MVK activity. V377I is frequently found in a compound heterozygous state in most MKD patients (5, 31). At the opposite hand, some variants (i.e., V310M, A334T) are closely associated with a severe MA phenotype and severely impaired cellular MVK activity (32).

Interestingly, the H20P and I268T mutations have been described in intermediate phenotype, either with MA and MKD clinical signs, such as fever attacks associated with some neurological manifestations (mental retardation, cerebellar ataxia) of variable severity (33), leading to the hypothesis that the two diseases may represent the two extremities of the phenotypic spectrum which depends on the type (truncating vs non-truncating) of the mutations or the degree of impairment of the MVK enzyme activity. For example, mutations resulting into a MKD phenotype are exclusively missense, associated with a mild reduction of the enzymatic activity whereas in the MA phenotypes, frameshift and nonsense mutations are commonly reported (16), which completely inactivate the gene function. In fact, the *MVK* gene encodes the enzyme MVK, involved in the ATP-dependent phosphorylation of mevalonic acid into 5-phosphomevalonate. Mutations affecting this gene alter the MVK activity, with an overproduction of proinflammatory isoprenoids, reduced synthesis of cholesterol, and accumulation of mevalonic acid in plasma and urine. Fever rushes may be caused by high release of IL-1β as a consequence of insufficient geranylgeranyl pyrophosphate generation (32). The development of fever may be caused by a dysregulation of the MVK pathway, but the pathogenetic mechanisms leading to the autoinflammation remain to be clarified.

### Tumor Necrosis Factor Receptor-Associated Periodic Syndrome

Tumor necrosis factor receptor-associated periodic syndrome (OMIM 142680) is the most common autosomal dominant AID in Europe; it was initially named “familial Hibernian fever” from the ancient Latin name “Hibernia” given to Ireland. In fact, in 1982, a large family from Scotland and Ireland was described with a new disorder, characterized by recurrent fevers, skin rashes, monocytic fasciitis, and abdominal pain (2). Since the first description, several cases have been identified in many other populations, such as Black Americans, Japanese, and patients of Mediterranean ancestry (34).

In 1998, the genetic basis of this condition was discovered and the name became TRAPS due to its relationship with the p551A receptor of TNF (TNFR1), encoded by the TNF super family receptor 1A (*TNFRSF1A*) gene (1, 2), whose mutations cause the disease. The *TNFRSF1A* gene is composed of 10 exons with the disease causing mutations, all missense and heterozygous, all concentrated into exons 2, 3, 4, and 6 (2). Based on the mutation position, they can be distinguished as high- or low-penetrance missense mutations. The high-penetrance mutations are located in cysteine-rich N-terminal domains, which are important for the assembly of the receptor’s three-dimensional structure (35, 36); furthermore, they cause an early disease onset and more severe clinical manifestations; the substitutions result in single amino acid substitutions in the cysteine rich domains (CRDs) 1, 2, or 3 of the ectodomain of the mature TNFR protein (37). These CRDs are involved in disulfide bond formation and in the folding of the extracellular portion of the protein. On the other hand, the *TNFRSF1A* low-penetrance mutations, such as R92Q and P46L, are associated with lower risk of amyloidosis and adult-onset, milder and/or atypical clinical features (16, 38–40); for instance, the P46L substitution occurs in up to 20% of clinically asymptomatic West African individuals, which suggests that it represents a polymorphism rather than a disease-causing mutation, whereas the R92Q substitution, relatively common in the Caucasian population, is a low-penetrance variant, which could have a weak contribution to disease expression. Moreover, *TNFRSF1A* mutations affecting TNF receptor shedding from cell membranes might potentially generate a selective advantage related to an increased antibacterial capacity (41). More in general, it can be assumed that, on the contrary to other fully penetrant autosomal dominant disorders, like neurofibromatosis type I (OMIM #162200), in which the new cases of the disease are all caused by new mutations in the *NF1* gene, the new cases of TRAPS belong to families in which the mutation segregates through the generations without giving manifest signs of disease. For this reason, in each proband, a careful family history for the cardinal signs of recurrent fevers, fasciitis, and cutaneous rash should always be collected.

Based on the difficulties with a clinical diagnosis of AID, a genetic test is useful in case of patients with clinical TRAPS phenotype, and a genetic diagnosis of TRAPS can be performed in the presence of a mutation in *TNFSRF1A*. Routine diagnostic analysis is limited to the exons 2, 3, 4, and 6, whereas the expansion of the analysis to the remaining coding regions of the gene is recommended only for cases with extremely suggestive phenotypes yet without a definitive diagnosis. In the absence of a family history and with borderline phenotypes, the probability of mutation finding with the extension of the genetic analysis to the whole gene remains very low and the decision should be carefully discussed with the clinician.

At pathogenic level, several mechanisms may be responsible for the disease onset, such as impaired TNF receptor shedding, defective intracellular TNF receptor trafficking to the cell surface, and subverted TNF-independent cell activation with increased production of IL-1 and IL-6, altered NF-κB pathway, increased activation of mitogen-activated protein kinases, and upregulated production of reactive oxygen species (42, 43).

The molecular link between TRAPS and IL-1 is not clear: the pathogenesis may vary with each mutation, but it is possible that IL-1 might act as a proinflammatory mediator downstream of TNF, or that aggregates of misfolded TNF receptors stimulate intracellular signals resulting in enhanced production of IL-1 and other chemokines (44).

### Cryopyrinopathies (FCAS, MWS, and NOMID)

Familial cold autoinflammatory syndrome (OMIM 120100), MWS (OMIM 191900), and CINCA syndrome (OMIM 607115), also known as neonatal onset multisystem inflammatory disease (NOMID), are autosomal dominant disorders (45–47) caused by mutations in the *NLRP3* (NOD-like receptor 3, cold-induced autoinflammatory syndrome 1, also named *CIAS1*) gene, encoding for the cryopyrin protein, an important inflammasome protein that directly activates IL-1β (48). Until 2001, these diseases were considered as three different diseases. Since 2001, mutations in the NACHT domain of the *NLPR3*/*CIAS1* gene were linked to FCAS and MWS (49, 50), whereas mutations in the same gene were identified in 2002 in sporadic cases with NOMID/CINCA (48, 51); after these evidences, the three disorders were grouped under the family of CAPS. FCAS and MWS are usually familial (49), while NOMID/CINCA is sporadic (52, 53).

Cryopyrin-associated periodic syndromes are rare diseases, with an estimated prevalence of approximately 1–2 patients per 1,000,000 people in Europe and in the USA (54).

Familial cold autoinflammatory syndrome, MWS, and NOMID/CINCA share a significant symptom overlap (55), with the latter described across the world as the most severe expression of CAPS (56).

In Infevers database, 175 different nucleotide variants and more than 90 heterozygous mutations on the *NLRP3* gene have been described to date (16, 57). Mutations in *NLRP3* gene are described in approximately 60% of CAPS patients, causing the constitutive activation of the inflammasome and dysregulation with IL-1 overproduction; excessive IL-1 signaling appears to be a constant feature in the background of CAPS, driven by gain-of-function *NLRP3* mutations, even in the absence of a second signal (58). As in all the AIDs, genetic testing is confirmatory, even if the diagnosis needs to be made on clinical symptoms.

More than 40% of NOMID/CINCA patients and a less percentage of FCAS and MWS patients do not carry germ-line mutations in *NLRP3*. In those patients, somatic mosaicism occurring during fetal development may explain the variation in disease onset (59); this mechanism can only be demonstrated by cell cloning and next-generation sequencing (NGS).

Genotype–phenotype correlations were demonstrated in CAPS, with some mutations associated only with a mild clinical phenotype, and others with severe clinical pictures. However, several cases were reported in which patients with the same mutations present different phenotypes (60, 61). Furthermore, some *NLRP3* mutations are described in healthy subjects with no signs of CAPS, such as V198M and Q703K genetic variants, even if there is no apparent selective advantage demonstrated for CAPS (62). Nevertheless, when patients who carry these polymorphisms show CAPS symptoms, the IL-1β-inhibition response is diminished. As in the other AIDs, functional polymorphisms may be considered low penetrance mutations, able to influence the activity of the gene product (58, 63).

In conclusion, CAPS onset may be influenced by environmental factors and genetic determinants, which are also able to modulate the disease phenotype.

### Deficiency of Interleukin-1 Receptor Antagonist

Deficiency of IL-1 receptor antagonist is a recently described autosomal recessive disease due to mutations of *IL1RN* that lead to non-expression of the encoded protein, IL-1 RA, causing unopposed IL-1 receptor activation and increased response to IL-1α and IL-1β stimulation (64).

The disease was first described in 2009 in nine patients presented with sterile multifocal osteomyelitis, periostitis, and pustulosis since the neonatal period, without fever. *IL1RN* gene was sequenced in those DIRA patients (6): either homozygous for mutations in *IL1RN* or heterozygous parents were identified. A patient was homozygous for two nucleotides deletion (c.156_157delCA) that caused a frame-shift mutation named N52KfsX25, followed by the incorporation of 24 aberrant amino acids and a termination codon. Both parents were heterozygous carriers of the same mutation. In other patients, three were homozygous for a nonsense variant affecting the amino acid residue at position 77 (c.229G>T; p.E77X). Patients from a consanguineous Lebanese family were homozygous for a nonsense mutation (c.160C>T, p.Q54X). Patient 9, from Puerto Rico, was homozygous for a deletion of approximately 175 kb on chromosome 2q that includes six genes from a cluster of IL-1-related genes: IL1RN and the genes encoding IL-1 family, members 9 (*IL1F9*), 6 (*IL1F6*), 8 (*IL1F8*), 5 (*IL1F5*), and 10 (*IL1F10*). The *IL1RN* mutations are present in founder populations in Newfoundland, the Netherlands, Puerto Rico, and possibly Lebanon and further founder mutations have since been found in other populations (65). None of these mutations were found in DNA specimens obtained from a panel of 364 controls from the New York Cancer Project.

In 2011, two unrelated Brazilian patients whose clinical phenotype was consistent with the DIRA syndrome were described (66). Both were homozygous for the same 15-bp (in-frame) deletion on *IL1RN*. This novel mutation of *IL1RN* produces a protein that does not bind the IL-1 receptor, and thus lacks functional activity. The authors hypothesize that this variant is likely to be a possible founder mutation in the Brazilian population.

In 2012, a novel nonsense mutation (p.Q119X) in *IL1RN* gene was identified in two Turkish patients with consanguineous parents (67).

### Blau Syndrome (BS)

Blau syndrome is an autosomal dominant granulomatous inflammatory disease caused by mutations in the *NOD2/CARD15* gene. This gene is located on chromosome 16q12 and encodes the three domain cytosolic protein of almost 1000 amino acids, the nucleotide-binding oligomerization domain containing 2 (NOD2). The protein contains two N-terminal CARDs for downstream signaling through CARD–CARD interactions, a central nucleotide binding and oligomerization domain (NACHT) with ATPase activity, and nine C-terminal LRRs for pathogen-associated molecular patterns (68).

Mutations in the *NOD2/CARD15* gene cause alteration in single amino acids in the NOD2 protein, resulting in an overactive version, which may lead to abnormal inflammatory reaction.

More details on the pathogenic aspects of BS were obtained from the identification in four European families of three missense mutations in 2001. Two of these families shared the same mutation, encoding an amino acid substitution of arginine to tryptophan in position 334 (R334Q), one family had an R334W and another L469F substitution (69).

The following year, another study on the genetic analysis of NOD2 coding regions based on 10 families with BS was published (70). In five of the families, two sequence variants at position 334 of the gene product (R334W and R334Q) were identified. Affected family members from the original BS kindred, included in this study, were heterozygous for the R334W missense mutation; mutations at the same position were also observed in several unrelated BS families, some of whose phenotypes included large-vessel arteritis and cranial neuropathy. The missense mutations were segregated with the disease phenotype in the families and were not identified in 104 healthy controls.

To date, on a total amount of almost 220 patients with BS carrying CARD15/NOD2 mutations, missense substitutions of R334Q/R334W account for more than 80%, causing a genetic hot spot for mutations in codon 334. E383K has been found in almost 5% of patients, whereas other mutations have been described most rarely (71).

The number of NOD2 variants associated with BS has expanded greatly. In fact, up to 2016, the number of sequence variants of *NOD2* gene is 144 (140 substitutions, 3 deletions, and 1 insertion) (16). There are no known mutations involving the untranslated and the intronic regions of the gene, even though this has not been extensively studied.

Despite the striking clinical similarities between them, for many years BS was considered a distinct entity from early onset sarcoidosis (EOS). Genetic analyses showed that many patients with EOS carry mutations in *CARD15/NOD2* gene; hereafter, some authors proposed that BS and EOS are the familial and sporadic forms of the same disease (71). Moreover, *CARD15/NOD2* gene mutations described in BS and EOS are mainly located in the NACHT domain of the protein. The discovery of *CARD15* mutations in BS families encouraged to investigate similar *CARD15* mutations in EOS patients.

Among 10 EOS cases retrospectively collected in Japan, heterozygous missense mutations were found in nine cases; four showed a c.1000C>T (p.R334W in amino acid change) that has been reported in BS, four showed novel c.1487A>T (p.H496L), c.1538T>C (p.M513T), c.1813A>C (p.T605P), and c.2010C>A (p.N670K), and one case showed double c.1146C>G (p.D382E)/c.1834G>A (p.A612T) mutations on different alleles. The study concluded that EOS is closely related with *CARD15* mutations causing constitutive NF-B activation and shares the common genetic etiology with BS (72).

### Deficiency of the IL-36 Receptor Antagonist (DITRA)

Deficiency of the IL-36 receptor antagonist is a recently described autosomal recessive autoinflammatory syndrome caused by mutations in the *IL36RN* gene, characterized clinically by recurrent episodes of generalized skin pustulation, fever, systemic inflammation, and leukocytosis. Other phenotypes of the *IL36RN* mutation include related pustular disorders, palmoplantar pustulosis, acrodermatitis continua of Hallopeau (ACH), and acute generalized exanthematous pustulosis. Histology shows spongiform pustules, acanthosis, and parakeratosis and an abundance of CD3+ and CD8+ T cells and macrophages (73).

This gene encodes IL-36 receptor antagonist (IL-36Ra), a protein belonging to the IL-1 cytokine family responsible for the tight regulation of IL-36 signaling. The IL-36 pathway is activated after binding of one of the three IL-36 agonists (IL-36b α, β, and γ) to a common specific receptor IL-1Rrp2, leading to the recruitment of the co-receptor IL-1 receptor accessory protein (IL-1RacP) and subsequent activation of intracellular NF-κB and mitogen activated protein kinase pathways (74).

Several mutations were highlighted in *IL36RN* gene with different effects. All *IL36RN* null mutations, such as c.28C>T (p.Arg10X), c.41C>A (p.Ser14X), c.80T>C (p.Leu27Pro), c.227C>T (p.Pro76Leu), c.280G>T (p.Glu94X), c.368C>G (p.Thr123Arg), c.368C>T (p.Thr123Met), and c.420_426del (p.Gly141MetfsX29) were totally unable to antagonize the IL-36 mediated activation of the NF-κB signaling pathway (75–83).

Among the most frequent genetic alterations is one Tunisian founder missense mutation: c.80C>T (p.Leu27Pro1); one European recurrent missense mutation: c.338C>T (p.Ser113Leu2); and one Japanese founder nonsense mutation: c.28C>T (p. Arg10*3).

The mutations c.95A>G (p.His32Arg), c.142C>T (p.Arg48Trp), and c.308C>T (p.Ser113Leu) only partly reduced the expression level of the corresponding IL-36Ra and consequently the capacity to repress the IL-36 mediated NF-κB signaling cascade. The detection of c.104A>G (p.Lys35Arg) and c.304C>T (p.Arg102Trp) mutations do not produce evident alterations in either protein expression and function, raising doubt about the actual pathogenic contribution of these genetic variants. They are classified as damaging by the pathogenicity prediction tools, such as SIFT and/or PolyPhen. For these mutations, additional functional studies are warranted to understand whether these variants truly have an effect on disease development or if they are polymorphisms (74).

Recently, Cordoro et al. showed a homozygous mutation within the *IL36RN* gene at position c.115+6T>C in a male adolescent with generalized pustular psoriasis (GPP) since infancy. This mutation has been shown to lead to a splicing defect resulting in exon skipping and a premature stop codon, leading to a truncated IL36Ra protein (81).

In summary, the c.28C>T (p.Arg10X) and c.115+6T>C (p.Arg10ArgfsX1) transitions are known to be founder mutations in cases reported in Japan (84). The c.115+6T>C transition is also recurrently found in Chinese and Malaysian patients (81, 85). The c.80C>T (p.Leu27Pro) transition is a recurrent mutation in Africa, and the c.338C>T (p.Ser113Leu) transition is a recurrent mutation in Europe (79–81). In contrast, the c.368C>T and c.368C>G transitions have been reported in one case in Japan (75, 76), the c.104A>G (p.Lys35Arg), c.142C>T (p.Arg48Trp), and c.304C>T (p.Arg102Trp) have been reported in one and two cases in Europe, respectively.

More in general, the null mutations are consistently associated with the more severe phenotypes of GPP and acute exanthematous generalized pustulosis (86, 87), whereas the hypomorphic alleles usually show a milder phenotypic expression featured by localized variants of palmoplantar pustular psoriasis (PPP) and ACH, although generalized pustular phenotypes can be observed as well in carriers of mild variants. The detection of the same gene variants in generalized and localized pustular phenotypes suggests the pathophysiological contribution of other factors such as disease-modifying genes, environmental factors, and epigenetic events, which may all influence disease onset, expression, and severity.

### Chronic Atypical Neutrophilic Dermatosis with Lipodystrophy and Elevated Temperature (CANDLE) Syndrome

Chronic atypical neutrophilic dermatosis with lipodystrophy and elevated temperature syndrome is a rare autosomal recessive AID, with less than 100 cases described worldwide (88).

The CANDLE syndrome is caused, in the majority of cases, by homozygous mutations in PSMB8 gene, which encodes for a proteasome protein (89). There are evidences that an increase of modified and oxidated proteins occurring in fat and tissue cells, due to mutations of PSMB8 lead to an augmentation of cellular stress and apoptosis (88).

In 2012, a genome-wide analysis of nine CANDLE syndrome affected patients in eight families suggested that mutations in *PSMB8* gene may cause the CANDLE syndrome. In this cohort, four patients were homozygous and two were heterozygous for a missense mutation (p.T75M), two patients were homozygous for a nonsense mutation (p.C135X), and one patient showed no mutations. None of these gene variants were observed in 750 healthy controls. Furthermore, only two of the four patients with the same mutation shared the same haplotype, indicating a possible mutational hot spot (90). Later, in 2015, a diagnosis of CANDLE syndrome was performed by targeted sequencing of one 3-year-old CANDLE syndrome Hispanic male patient, born to consanguineous healthy parents. A homozygous c.280G>C, p.A94P mutation in *PSMB8* gene was not observed in any public genetic databases and was predicted to be pathogenic by several prediction tools (91).

Recently, it was demonstrated that CANDLE syndrome can also be caused by mutations in genes that encode other proteasome subunits, such as PSMB4, PSMB9, and PSMA3 (92); these mutations affect transcription, protein expression, protein folding, proteasome assembly, and, eventually, proteasome activity.

### Majeed Syndrome

Majeed syndrome is a congenital predisposition to develop early-onset multifocal osteomyelitis (CRMO), congenital dyserythropoietic anemia (CDA), congenital anemia, and inflammatory dermatosis, resulting from the infiltration of neutrophils into the dermis (93). It is transmitted with an autosomal recessive pattern of inheritance and is caused by mutations in the *LPIN2* gene, which maps on chromosome 18p. The genomic sequence of *LPIN2* is approximately 95 kb and comprises 20 exons, of which exon 1 and the majority of exon 20 are non-coding (5′ and 3′ untranslated regions). The mRNA is approximately 6245 bp and encodes a protein of 896 amino acids, which is expressed in almost all tissues (94). LIPIN2 derives its name from its highly conserved N-terminal and C-terminal LIP domains. LIPIN-1, -2, and -3 are phosphatidate phosphatases (PAPs), which are important in glycerolipid biosynthesis and as transcription co-activators regulating lipid metabolism genes (95). In addition, lipin-2 regulates increased IL-1β formation in primary human and mouse macrophages by several mechanisms, including activation of the inflammasome NLRP3. In macrophages, reduced levels of lipin-2 cause a decrease of cell cholesterol levels. In conclusion, lipin-2 is able to down-regulate NLRP3 inflammasome (91).

Several mutations have been identified in *LPIN2* gene. One of them, the c.540-541delAT (p.Cys181Ter), is a frameshift mutation that produces a premature stop codon producing a truncated protein that is 180 amino acids long; a second variant, the c. 2201C>T (p. Ser734Leu), is a missense mutation that replaces a highly conserved serine with a leucine (96). Al-Mosawi et al. report a third unique mutation in *LPIN2* in an Arabic female with CRMO and CDA. The c. 2327+1G>C (p.Arg776SerfsTer66) nucleotide change affects a highly conserved nucleotide residue at the 5′ (donor) splice site of exon 17 and it is predicted to introduce a frameshift mutation resulting in a premature stop codon being encountered in intron 17, which would be predicted to produce a truncated message (97). A novel homozygous 2 bp deletion (c.1312_1313delCT) resulting in a premature stop codon (p.Leu438fs+16Ter) and consequently in a truncated LIPIN2 protein was recently described in two Turkish brothers with MS who were treated successfully with IL-1 inhibitors (7).

*LPIN2* shares homology with *LPIN1*, which has been shown to play a role in murine lipodystrophy (98). The role of *LPIN2* mutations in producing the inflammatory phenotype of MS is not clear and does not appear to involve a disturbance in lipid metabolism. *LPIN2* has an amino-terminal lipin domain, a Lipin/Ned1/Smp2 domain, and a putative nuclear localization signal. Lipin2 also has PAP type-1 activity and may play a role in lipid biology (99).

Although the number of individuals reported with MS is too small to study genotype–phenotype correlations, the affected individuals with a frameshift variant appear to have a more severe course and complications than individuals with other classes of pathogenic variants (100). More recent observations, however, have indicated that an affected individual with a splice site variant (97) and two affected Turkish brothers with a frameshift variant (7), who were all diagnosed and treated early, had a less complicated course. It is unclear whether their milder clinical course is attributable to the earlier detection and treatment.

### CARD14-Mediated Pustular Psoriasis (CAMPS)

*CARD14* encodes caspase recruitment domain family member 14 (CARD14). It is known to be specifically expressed in the skin and to be localized mainly to keratinocytes. CARD14 is a scaffolding protein that regulates NF-κB activation. The NF-κB family of transcription factors plays a crucial role in cell activation, survival, and proliferation and results in cancer, immunodeficiency, or autoimmune disorders (e.g., psoriasis). Hence, the presence of the CARD14 mutations may result in greater amplitude of inflammatory response upon epidermal activation. The skin disease in patients with *CARD14* mutations can be limited or generalized. Autosomal dominant or sporadic gain-of-function mutations in the *CARD14* gene cause GPP (101), familial pityriasis rubra pilaris (PRP) (102), psoriatic arthritis (PA) (103), PPP (104), and even pustular psoriasis suggesting a large disease severity spectrum. Fever and other systemic manifestations are generally not present but can occur with superinfections of the skin.

Three variants, c.349G>A (p.Gly117Ser), c.205C>T (p.Arg69Trp), and c.589G>A (p.Glu197Lys), affect the N-terminal region of the protein harboring its caspase recruitment domain or coiled-coil domain but with different effects. The c.589G>A (p.Glu197Lys) and c.349G>A (p.Gly117Ser) lead to upregulation of NF-κB activity, whereas the c.205C>T (p.Arg69Trp) leads to a sevenfold downregulation. In particular, the c.349G>A (p.Gly117Ser) variant described in a family of European descent altered the splicing between *CARD14* exons 3 and 4. One Tunisian patient was reported with a c.1356+5G>A splice alteration which is predicted to lead to the skipping of exon 9, which encodes part of the coiled-coil domain (105). Mutations in *CARD14*, including p.Glu138del and p.Leu156Pro, have been associated with autosomal-dominant pityriasis rubra pilaris, which is phenotypically related to psoriasis (102).

Several gain-of-function variants/mutations in *CARD14* have been reported to be a predisposing factor for psoriasis vulgaris (PV) in a large family with PV and PA. Jordan et al. identified the rare *de novo CARD14* gain-of-function variant p.Glu138Ala in a child with severe early-onset GPP. They also found rare *CARD14* gain-of-function variants in large PV cohorts by the NF-κB assay, which revealed that compared to the wild-type CARD14, the p.Gly117Ser, p.Glu138Ala, and p.Asp176His variants were associated with increased levels of the luciferase reporter. Additional rare variants within CARD14 are c.424G>A (p.Glu142Lys), c.511C>A (p.His171Asn), c.536G>A (p.Arg179His), and c.571G>T (p.Val191Leu) (106).

In conclusion, the above-reported data concur to reach the following conclusions: (1) differences in the genetic background among different geographic populations account for significant variances observed in psoriasis populations, both in terms of frequency and of severity (localized palmoplantar versus generalized forms) of pustular psoriasis (107, 108), (2) despite the dramatic *in vitro* effects of some CARD14 variants on the keratinocytes, there is a wide range of phenotypes, even among individuals who carry the same substitution, suggesting that in many instances, the variable phenotypes are likely to multiple other factors besides the genetic background.

### NLRP12-AID

NLRP12 is an NLR encoded by *NLRP12* (also known as *NALP12*, Monarch-1, or PYPAF7) and functions as a negative regulator of NF-κB activation (109). NLRP12 interacts via its pyrin domain with the pyrin domain of apoptosis-associated speck-like (ASC) protein (110) leading to the formation of an intracellular aggregate called speck to active IL-1B (111). Sequencing of *NLRP12* revealed a heterozygous nonsense mutation c.850C>T (p.Arg284X) in identical twin brothers presenting with symptoms overlapping FCAS and MWS. A second NLRP12 mutation (c.2072+3insT) was identified in a patient presenting with a periodic fever syndrome, including clinical manifestation of FCAS. This mutation, affecting the donor splice site of intron 3, activates a cryptic splice site located upstream in exon 3 and results in a frameshift, followed by a premature stop codon (112). These two mutations were demonstrated to be functionally associated with high levels of NF-κB activity, thus accounting for the autoinflammatory phenotype. Jèru et al. identified a missense mutation c.1054C_T (p.Arg352Cys) within the NBS domain of the protein in two unrelated patients. This missense mutation is associated with a gain of function of caspase 1 processing (110). The c.882C>G (p.Asp294Glu) mutation was found to mostly segregate with a particular sensitivity to cold exposure (especially arthralgias and myalgia), even in the absence of urticarial rash, fever, or elevation in the levels of acute-phase reactants. In any case, the clinical manifestations presented by the carriers were generally mild, although quality of life was affected, especially during the winter season (112–114). Several reports identified the NLRP12 variant F402L (c.1206 C>G) (115–117).

### Pyogenic Arthritis, Pyoderma Gangrenosum, and Acne (PAPA) Syndrome

Pyogenic sterile arthritis, pyoderma gangrenosum, and acne syndrome is an autosomal dominant AID caused by mutations in the *PSTPIP1* gene, which is located in chromosomal position 15q24–q25.1. Pyrin protein is a cytosolic receptor for PSTPIP1. Ligation between pyrin and PSTPIP1 induces pyrin to interact with ASC protein, inducing the creation of an active ASC pyroptosome. A possible explanation of PAPA syndrome is a constitutive ligation and consequent activation of pyrin with the mutated PSTPIP1 proteins (112). The disease is extremely rare, with less than 10 families described worldwide. The first case of PAPA syndrome was reported in 1975 (118) and in 1997, in the same family, PAPA syndrome was described as a heritable disease (119).

In 2000, 93 genomic loci were investigated in patients with a pleiotropic inflammatory syndrome characterized by pyoderma gangrenosum, cystic acne, and erosive arthritis, demonstrating PAPA syndrome maps in chromosomal position 15q (120).

There are two hot-spots mutations, c.688G>A (p.A230T) and c.748G>C (p.E250Q), which occur in exons 10 and 11 and have been found in many familial (121–126) and sporadic cases (127, 128). Mutations are thought to disrupt the binding of PSTPIP-1 with protein tyrosine phosphatase–PEST, a regulatory phosphatase, increasing its avidity for pyrin in the cytosol, thereby causing dysregulation of IL-1β production (129).

Until 2016, a total of 27 genetic variants were reported for *PSTPIP1* gene (23 substitutions, 1 insertion, 2 deletions, and 1 duplication), 17 of which are PAPA phenotype associated (16).

### Deficiency of Adenosine Deaminase 2

Searching for mutations in systemic inflammation and vasculopathy and/or necrotizing vasculitis polyarteritis nodosa patients, *CECR1* (cat eye syndrome chromosome region, candidate 1) gene mutations were discovered by two independent groups. Pattern of inheritance was autosomal recessive. Subsequent studies described another case with a fatal vasculopathy (130). Common clinical signs are early onset recurrent stroke, neurologic manifestations, and fever. Being the *CECR1* gene highly polymorphic, as other AID causative genes, the correlation between clinical signs and familial ancestry is important. The *CECR1* gene encodes the adenosine deaminase 2 (ADA2) protein, which has partial homology with ADA1 protein. Both ADA1 and ADA2 act as intracellular enzymes that regulate the purinergic signaling pathway. Mutations in ADA1 are known to cause severe combined immunodeficiency disease, characterized by a defect in T- and B-lymphocytes. ADA2 mutations, conversely, cause only mild hypogammaglobulinemia due to a defect in terminal differentiation of B-cells (131).

### NLRC4-Associated Inflammatory Diseases (SCAN4, NLRC4-MAS, and NLRC4-FCAS)

Patients with macrophage activation syndrome or a milder phenotype like FCAS may carry gain of function mutations in the *NLRC4* (IPAF; CARD12) gene (132–134). Two novel causal mutations, p.T337S and p.V341A, have been diagnosed by whole exome sequencing (WES) in two sporadic patients (trios); the reported clinical symptoms were early onset fever, failure to thrive, rash, joint pain, and elevated inflammatory markers, including hyperferritinemia (133). The two mutations map in a highly conserved HD1 region of the NLRC4 nucleotide-binding domain and may decrease the function of NLRC4 to maintain itself in an auto-inhibited state. A third mutation, p.H443P, was identified in a Japanese family with milder symptoms including cold induced rash, fever, and arthralgia, even if the patient has a different phenotype from those other two previously patients reported. Mutations are supposed to regulate the mutant proteins into a constitutively active state, with the influence of environmental factors such as cold and stress which act as trigger, causing inflammasome activation. Further studies are necessary to explore full spectrum of monogenic inflammasome-related diseases. Interestingly, the *NLRC4* gene (IPAF, CARD12) is supposed to initiate inflammation in response to bacterial ligands, such as flagellin (135, 136).

### Stimulator of Interferon Genes (STING)-Associated Vasculopathy with Onset in Infancy

In a trio with a patient affected by early onset symptoms of systemic inflammation, cutaneous rash, and pulmonary manifestations, and his unaffected parents, WES was performed, identifying a *de novo* mutation, p.N154S, in the TMEM173 gene; this gene encodes for a STING (138). Extending the analysis to other five sporadic cases of different ancestries but with similar phenotype led to the identification of missense mutations in the TMEM173 gene. Functional studies showed that a particular missense mutation, p.V155M, has been previously associated with a phenotypically different disease like systemic lupus erythematosus (139). Most of the mutations are located in the exon 5 of TMEM173 gene, which encodes for a domain important for the STING dimerization site. The second study highlights that in families with dominantly inherited traits, the possibility of reduced penetrance should not be ignored. Sting knockout mice are prone to viral infections, because of the lacking of the ability to upregulate IFN-beta (137). As a result, all these data support the evidence that TMEM173-associated mutations are gain of function (138, 139).

### TNFRSF11A-Associated Hereditary Fever Disease

Patients with this disease have a phenotype similar to those affected by TRAPS. In a single patient with complex phenotype including neonatal onset of systemic inflammation and congenital abnormalities, a *de novo* genomic duplication containing the TNFRSF11A gene was identified (140). The TNFRSF11A gene is one of the 30 genes in the 10-Mb genomic duplication. Another study, using a different approach based on candidate gene screening, identified two other patients (mother and daughter) with a novel heterozygous 1-bp deletion (p.Met416Cysfs*110) in exon 9 of *TNFRSF11A* gene. The resulting protein lacks the C-terminal intracellular domain. As TNFRSF1A, also TNFRSF11A gene encodes for a protein member of the TNF-receptor superfamily. *TNFRSF11A* (RANK, PDB, ODFR) gene encodes a signaling receptor that functions in osteoclast differentiation and bone remodeling (141, 142). RANK-ligand (RANKL) mediates essential signal for osteclastogenesis (143).

Rank-deficient mice present osteopetrosis caused from a block in osteoclast differentiation and the lack of peripheral lymph nodes (144). The pathogenesis of this disorder is unclear; in fact, the *TNFRSF11A* gene duplication suggests a gain of function mutation, while the heterozygous deletion is more consistent with a haploinsufficiency or a dominant negative effect.

### TRNT1 Deficiency

Patients presenting with a variable phenotype of congenital sideroblastic anemia, B cell immunodeficiency, and developmental delay have been termed SIFD (145). SIFD is characterized by an early onset, with frequently associated neurological symptoms, and metabolic abnormalities. SIFD has been associated to AID because it is characterized with a pediatric onset with periodic fevers and gastrointestinal involvement with sideroblastic anemia. Genetic cause of SIFD has been found in the *TRNT1* gene in an autosomal recessive inheritance (146, 147). The *TRNT1* gene encodes the ubiquitously expressed CCA-adding enzyme, essential for template-independent maturation of nuclear and mitochondrial transfer RNAs (148). Functional studies in yeast showed deficiency of TRNT1 homolog causes partial loss of function of TRNT1 affecting variable degrees of enzyme activity (146). Knock-out yeast for *TRNT1* was fully restored with human TRNT1 and partially rescued by human mutant proteins.

### Monogenic Form of Systemic Juvenile Idiopathic Arthritis

Systemic-onset juvenile idiopathic arthritis is a polygenic inflammatory disease characterized by fever, rash, and symmetrical polyarthritis, with persistent systemic inflammation that seems to be linked to altered innate immune system (149, 150). Mendelian inheritance is autosomal recessive. Studying five consanguineous families with 13 affected patients from the Saudi Arabia with several genomic approaches, such as linkage analysis, homozygosity mapping, and WES, a homozygous missense mutation, p.C284R, located in exon 4 of the laccase domain containing 1 (LACC1) gene was identified (151). This private mutation is highly conserved during evolutionary scale and was not described in more than 2,000 Arab controls, suggesting its role in the pathogenesis.

Laccase domain containing 1 gene belongs to a family of Laccases, multi-copper oxidoreductases able to catalyze the oxidation of a variety of phenolic and non-phenolic compounds. Protein function is largely unknown, even if may regulate the innate immune responses. In a recent study, the gene product of LACC1 gene has been named FAMIN (fatty acid metabolism–immunity nexus), important for the synthesis of endogenous fatty acids and their mitochondrial oxidation, controlling the glycolytic activity and ATP regeneration (145).

Genetic variants in the LACC1 gene have been previously associated with susceptibility to leprosy (152–154). LACC1 gene belongs to a family of Laccases, multi-copper oxidoreductases able to catalyze the oxidation of a variety of phenolic and non-phenolic compounds. Protein function is largely unknown, even if may regulate the innate immune responses.

## Genetic Diagnosis with New Technologies

In recent years, with the increased use of instruments for NGS, it has become feasible to analyze several genes in a single experiment. This method is of great interest for AID, because of the increasing number of genes associated with the different forms of AID. In some cases, it is difficult to differentiate the different diseases, in particular, in those patients with intermediate phenotypes or a difficult clinical diagnosis. The analysis of a panel of several candidate genes is feasible with targeted sequencing investigating causative mutations, rare variants, or regions associated with the disease.

With the large number of genetic variants found with NGS, it is extremely important to find information about their possible role in the development of a specific disease. Several softwares have been developed in recent years and most of them have been based on the assumption that protein sequences derived from living organisms have survived a natural selection. The goal is to find if a genetic variant is a causative mutation or a common polymorphism.

There is also the possibility to analyze all the 18,000 genes of the human genome. WES is a recent strategy designed to sequence only the coding regions of the genome (which represents 1% of the human genome, about 30 megabases); this is an effective method alternative to whole genome sequencing, cheaper and less complicated in the bioinformatical/statistical analysis. Exons are generally short, functional sequences of DNA which represent the portion of the genome translated in protein. The WES has the potential to identify the coding variants responsible for both Mendelian and common diseases.

In recent years, with the use of WES, several undiagnosed Mendelian genetic conditions have been investigated in order to search the involved gene (155, 156). WES has been applied to the study of trios (unaffected parents and the sporadic case) and unrelated patients with phenotypic similarities. Identification of causal gene in a single sporadic patient can then be confirmed in other patients with similar phenotypes. The potential of such strategies is high in polygenic human disorders such as AID. The emerging genetic technologies complemented by the development of public databases of human variation can allow discovery of disease causal genes in sporadic and unrelated patients.

Whole genome sequencing allows to sequencing the entire genomic DNA, both chromosomal and mitochondrial. Unlike WES, this method allows the sequencing of both exons and introns, for a total amount of 3 Gigabases.

In a single center study (157), more than 2,000 diagnostic patients have been analyzed with Sanger sequencing for the NLRP3, MVK and TNFRSF1A genes, and other AID gene portions, failing to find mutations in 86% of samples. Possible explanations of this high failure are the restricted number of tested genes, clinical misdiagnosis, genetic heterogeneity, and/or a complex mode of inheritance. In order to improve the sensitivity of the genetic tests, 50 patients were re-analyzed with a gene panel of 10 genes for NGS procedure. The 10 genes were *MEFV, MVK, TNFRSF1A, NLRP3, NLRP12, NOD2, PSTPIP1, IL1RN, LPIN2*, and *PSMB8* (157). In order to better understand the possible role of the detected variants, allele frequencies have been compared with those of 1,000 Genomes Project, and searching for a possible genotype–phenotype correlation. Some genes, such as *NOD2, LPIN2*, and *NLRP12*, showed a high frequency of genetic variants, which in theory may alter the clinical phenotype with mild or atypical symptoms. In the next future, NGS data combined with clinical information may help diagnosis for those patients with intermediate phenotype (156); in fact, the interaction between geneticists and physicians will allow to improve the diagnosis of AID patients (157).

## Conclusion

In AID, genetic and environmental factors act modulating the clinical presentation of a specific disorder. The knowledge of the biological pathways at the basis of different AIDs is very important; the elucidation of these novel factors may have clinical relevance, because it may be included in genetic-risk modeling approaches. The genetic variants previously identified as playing a role in the same pathway represent new potential therapeutic targets. The new age of the *-omics* has allowed the improvement of the knowledge of AID. By means of genetic fine mapping, targeted sequencing, transcriptomics, proteomics, and metabolomics, physicians may improve treatment and therapy tailored on the single patient.

## Author Contributions

DM contributed in conception or design of the work, drafting the article, and final approval of the version to be published. FB and PM contributed in drafting the article and final approval of the version to be published. AV contributed in drafting the article, critical revision of the article, and final approval of the version to be published. AP contributed in conception or design of the work, drafting the article, critical revision of the article, and final approval of the version to be published.

## Conflict of Interest Statement

The authors declare that the research was conducted in the absence of any commercial or financial relationships that could be construed as a potential conflict of interest.
